# Operation for Circumcision—Apparatus for Aseptic Paracentesis Abdominis—Two Cases of Syphilitic Brain Lesion

**Published:** 1890-04

**Authors:** Howard J. Williams

**Affiliations:** Macon, Ga.


### OPERATION FOR CIRCUMCISION—APPARATUS FOR ASEPTIC PARACENTESIS ABDOMINIS—TWO CASES OF SYPHILITIC BRAIN LESION.

REPORT ON SURGERY READ BEFORE THE MACON MEDICAL SOCIETY, SEPTEMBER 17th, 1889.

BY HOWARD J. WILLIAMS, M. D.

I wish to describe the following method of operating for phimosis, as I find it does away with many of the unpleasant attendants on circumcision.

At the time of operation, the patient is required to wash the penis and adjacent parts, first with soap and water, then with bichloride solution (1 to 1,000); care is taken to thoroughly cleanse the mucous membrane around the corona glandis. To obtain local anæsthesia, cocaine is used in the following manner: The needle of the hypodermic syringe, containing a solution of muriate of cocaine (5 grains to 100 drops of water), is introduced into the connective tissue on the dorsum of the penis, and is made to pass between the skin and mucous membrane of the fore-skin, along a line corresponding to the corona glandis, until the frenum on one side is reached. The needle is now gradually withdrawn, and the cocaine is deposited as the instrument is being retracted. This process is repeated on the other side of the penis. From fifteen to twenty drops of the solution is thus deposited, and a complete circle of cocainized tissue is obtained. This circle will be used as the field of operation.

A narrow rubber band (such as is used around small packages), made aseptic by the bichloride bath, is now placed around the base of the organ to act as a tourniquet. If the band is applied before the cocaine injection, the loose skin of the penis is apt to be drawn by the band back towords the pubis; and, when the cocaine is injected, the line left by the hypodermic needle will not be in the tissues overlying the corona glandis, but in the tissues of the fore-skin nearer the end of the penis, and as this line is used as the line of incision, too much fore-skin will be left after the operation.

In about five minutes the full effects of the cocaine will be obtained, and the next step of the operation can be taken. The circumcision forceps is applied, and with a sharp knife the fore-skin is cut off, the band of cocainized tissue being used as the line of incision. To the surprise of the patient there is no pain, and after a minute or two all bleeding stops. The anæsthesia is complete and the hemorrhage amounts to only a teaspoonful, consisting solely of that blood detained in the tissues by the constricting band. The forceps is removed and the mucous membrane left by the first incision is next trimmed to the proper extent. The stitches (the finest silk thoroughly aseptic I prefer) are introduced, from four to six, as the case may require, being used.

The wound is left open for a few moments, to allow the atmosphere to glaze over the raw surface, and so close all vessels likely to bleed when the tourniquet is removed. The stitches are then drawn up, the edges are approximated, and the wound is ready for the last step in the operation, namely, the application of a thick coating of *iodoform collodion.* This application is made with a camel's-hair pencil, and should extend from a half to an inch on either side of the edges of the wound. I formerly incorporated in the iodoform dressing some fibres of cotton to strengthen the dressing, but now use the iodoform collodion alone, finding that it is sufficiently strong of itself and much easier of removal. The band is removed as soon as the collodion is dry, and the operation is completed. In five or six days when the collodion is ready to come off, and the stitches are removed, it will be found that primary union has taken place

By the above method of operating, pain is avoided without the employment of general anæsthesia; hemorrhage is reduced to a minimum; the swelling is controlled by the collodion; a thoroughly antiseptic dressing is obtained, under which primary union can take place; the patient is not troubled with any after dressing, which formerly he had to renew frequently, and often with soiled hands which carried germs to the wound; the urine cannot get to the wound and irritate it; and the patient is not detained from his business, but continues his daily pursuits without discomfort. After the operation I always prescribe an antaphrodisiac, either paregoric, or what I prefer a pill of codeia sulphat. gr. i, extract. hyoscyamii gr. ss, and camphor gr. ii every three hours. Erections, while they are not apt to disturb the dressing, are extremely uncomfortable to the patient.

I should like to show you a simple device to be used in paracentesis abdominis, making the operation a thoroughly antiseptic one. This device I saw described some time since in a medical journal, and its utility so impressed me that I decided to employ it in the next case requiring paracentesis. It consists simply of an ordinary trocar and a long rubber tube. I have had the shoulder on this trocar moved forward, so that the rubber tube may be slipped on the end of the canula, back of the shoulder, as you see. Now, the tube is bent down thus, so as to present a flat surface against the end of the canula. A small puncture is made is this flat surface, and the stylet is pushed through it, into and through the canula. The whole apparatus is then submerged in a tub containing an antiseptic bath; the air in the tube escapes, and is replaced by the water. The trocar is now removed from the water, while the other end of the tube is kept submerged, and a column of water remains in the tube. When the trocar is plunged into the peritoneal cavity, the stylet is withdrawn from the canula through the opening in the tube, which closes and the fluid escapes from the abdominal cavity through the canula and rubber tube, and is discharged under the surface of the water in the tub. Some days ago I demonstrated the utility of this device to my own satisfaction, and I believe Dr. McHatton can indorse it, as he was with me at the time.

I report the two following cases of brain trouble, first because they are syphilitic, and again, because all brain troubles are now of surgical interest. Since cerebral localization has assumed, such prominence, and operative interference in most brain affections is now recognized as not only justifiable but often imperative, the consideration of these cases will not be without interest.

I. J. W., colored, aged 51 years, a laborer, born in Georgia, family history good. Twelve years ago (dating from the time of observation) he had inflammatory rheumatism. Eight years ago he had a chancre and bubo. From that time his health became impaired, his mind becoming dull, and one day he fell partially unconscious. Following this he was paralyzed, the right leg, arm, side of face and tongue being affected. Recovery after some months was slow and imperfect. His face remained slightly drawn to the left, articulation quite imperfect, the right arm wasted and almost useless, and the leg dragged when walking. In September, 1886, he came under observation, presenting in addition to the above history, the following train of symptoms: About six months before, he began to suffer with pain in his forehead, attended by slight vertigo and pains in the right shoulder. Three months later an enlargement on the frontal bone, in the median line about two inches above the nose, made its appearance and steadily increased in size. The tumor was painful on pressure, could be slightly indented, and seemed to be of soft consistence. The pain complained of was located in the tumor, was characteristic of bone lesion, subject to exacerbation and particularly severe at night. There was constant headache in addition, distinct from the pain in the bony mass, and described as a "pressure on the brain." He was troubled with constant tinnitus aurium and vertigo, which daily increased in severity. His mind was very dull and he had great difficulty in comprehending directions. Nausea and occasional vomiting were at times present. The pulse was slow and there was no increase of temperature. Much loss of flesh and strength. The treatment was bichloride of mercury and large doses of iodide of potassium. Rapid relief to the symptoms followed the treatment. The tumor diminished in size, the headache and pains were relieved, and there was an almost complete restoration of health. The loss of power on the right side following the old paralysis was of course unaffected by the treatment. He is still (1889) under observation, and as yet there is no sign of relapse.

The next case came under observation in 1887.

II. H—, white, aged 45 years, a machinist, born in Georgia, family history good, habits good, past history doubtful, denies syphilis, but was very dissipated when young.

April 20th, 1887, he came for treatment, complaining of neuralgia of some weeks' duration, attacks of pain in the head, vertigo, slight nausea, but no vomiting. Has had at times numbness in the left arm with tingling in the hand. His friends thought that his mind was impaired; some disposition to loquacity. There were several small lumps on the head over the right parietal bone near the sagittal suture, about midway between the anterior-superior and the posterior-superior angles. They were extremely painful on pressure, could be slightly indented when pressed upon, were immovable and apparently attached to the bone. Characteristic bone pains, increased at night, were present in these masses, and were distinct from the neuralgic headache of which he complained. He had not as yet suffered sufficiently to compel him to quit work, though he was weak and had lost flesh.

May 10th, feeling about as usual after the day's work, while sitting with some friends talking, he was seized with a convulsion. It began in the left hand and extended to the left leg, then became general. Consciousness was not lost. The convulsion continued only a few moments, but returned in about fifteen minutes. During the evening he had four in all, each preceded by a feeling of numbness. The convulsions were witnessed by a gentleman of intelligence, and were described minutely to me. Not finding me, another physician was called, and he administered a hypodermic of morphine and gave a large dose of calomel. I saw him May 11th, about twelve hours after the last convulsion. The mouth was drawn slightly to the right. No defect or irregularity of vision. The speech slightly thick. The grasp of the left hand much weaker than that of the right; foot and leg unaffected. Sensation to pain is more acute on the left than on the right side.

As my diagnosis previous to the convulsive seizures had been syphilitic gumma he was already taking specific treatment, but in small doses. In view of the alarming nature of the attacks I ordered large doses of iodide of potassium in combination with ordinary doses of bichloride of mercury. To allay the mental excitement a few doses of bromide were given.

May 13th. Still in bed. Sitting up causes vertigo and a disposition to turn to the left.

May 26th. Headache gone, other symptoms improved.

June 1st. At work. No unpleasant symptoms; tumors disappearing.

I lost sight of the case soon after this, but recently (1889) learned that he had continued treatment some time and was doing well.

These were cases of syphilitic disease of the cranial bones, gummatous tumors; the first with a clear history, the second no history acknowledged. Specific treatment equally benefited both, and in the second case confirmed the diagnosis. In both cases there were two sets of symptoms present; first symptoms of syphilitic disease of the bones, and second symptoms of cerebral irritation due to pressure. Considering the second set of symptoms, the two cases illustrate that different portions of the brain differ in the degree with which they bear interference. The anterior portion of the brain is extremely tolerant; tumors, foreign bodies, or accumulations of pus may press upon the frontal convolutions or invade their tissues without causing very serious symptoms, as in the first case. The middle convolutions on the other hand readily resent interference. In these convolutions, near the fissure of Rolando, are located the centers controlling the functions of the arm, leg and side of face. Any irritation here is resented, convulsive attacks follow and are most marked in the limb whose center is most irritated. In the second case the tumors on the skull correspond with this region of the brain; the convulsion began and was most marked in the arm. Another point, by the way, illustrated by the two cases, is that irritative lesions cause convulsive action, while destructive lesions are followed by paralysis and loss of power. The old lesion in the first was followed by loss of power of the same region that in the second case is convulsed.

The consideration of the question of treatment of these cases is of importance. The relief to the pressure symptoms was urgent. Modern surgery in its aggressiveness has entered the cranial cavity and relieved the brain of diseases hitherto considered incurable; tumors, foreign bodies, accumulations of pus, inflammatory deposits and even diseased centers are cut down upon and removed. In such cases as these I report, the relief from pressure demands the consideration of operative interference. The nature of the growths, causing the irritation of the brain, rendered them however amenable to therapeutics and until specific treatment had been tried and failed, any operative interference would not have been justifiable. Had the nature of the trouble been other than syphilis, an exostosis, accumulation of pus, or adhesion of the brain and dura for instance, an operation would have been justifiable. The treatment of the irritative symptoms resolves itself, then, into treatment of the syphilitic bones, which is the ordinary specific modified as the case demands. A few remarks in this connection, rather dogmatically expressed, and I will close. First, large doses of iodide of potassium are indicated when small ones fail and when urgent symptoms arise, as in the second case. Second, recent lesions attended by irritative symptoms and lesions where destruction has not advanced far, are benefited by treatment, but it is useless to expect restoration of function in old lesions, where cicatriced tissue has followed destructive metamorphosis, as is well illustrated in the first case. Third, moderate doses of bichloride or biniodide of mercury will materially aid the large doses of iodide of potassium. Fourth, iodide of potassium will relieve pain and cause sleep in syphilis, when opium or other anodynes will not. Fifth, it is well not to place too much confidence in patient's statements.

Dr. McHatton at the conclusion of this paper reported the following case of syphilitic brain lesion:

---

### CASE OF GUMMA OF THE BRAIN, REPORTED BY H. MCHATTON, M. D., MACON.

The report of the above cases of specific brain lesion by Dr. Williams calls to my mind one of my own that should be included with them. In October, 1885, I was called out of the city to see a gentleman of some standing; arriving at the place of meeting I found the family physician unavoidably detained. The patient, a man of some fifty years, was comatose, with the following history, obtained from his wife. Several months ago he began to complain of violent headache, confined almost exclusively to the motor center of the right arm, the pain increasing at night; dizziness and occasional nausea. He gradually lost the use of the right arm, and before coma came on paralysis of the member was complete, partial muscular atrophy having taken place, control of the leg on the same side being poor. The cerebral symptoms gradually increased until complete coma supervened four or five days before my arrival.

Present condition, coma absolute; liquids poured into the mouth are gradually swallowed, and that is the only evidence of sensibility. Action of bladder and bowels involuntary, breathing stertorous, pulse slow and full. Physical examination negative, examination of urine negative. Diagnosis, a growing tumor of the brain, possibly specific. On arrival of the family physician I was informed that twenty-two years ago the patient had a suspicious sore, but for the last eight or ten years no visible manifestation of the disease. The attending physician's diagnosis had been specific brain disease, but he was somewhat shaken on finding that he did not get the desired response to his remedies in the usual dose. After due consultation we decided that anti-specific medication afforded the only chance, and we began by the administration of pot. iod. in 30 gr. doses every three hours, to be continued or increased until death or improvement occurred.

The above dose was continued for a period of about ten days, gradual and constant improvement taking place all the time. The patient was then put on the mixed treatment with rather larger doses of pot. iod., with the advice to continue the treatment for at least two years. Present condition (Sept. '89), I am informed, is as follows. Paralysis of arm complete, control of leg perfect, mental faculties and general health good, he being at present at the head of rather large business enterprises. There are several points of interest in connection with this case. First, the location of the unquestionable gummatous deposit, which I think was in the center of the motor area of the arm, pushing towards that of the leg, the trouble with the latter being from pressure and not from deposit of new tissue. Of course we cannot tell how much of the center of motion of the face and aphasia may have been involved during the period of coma, as brain tumors of this region are usually painless; we have the pain to account for, which I think can be attributed to a local meningitis from pressure.

Second, the non-response to the usual doses of specific remedies, which is not as unusual as we would think. I often see lesions that do not respond to 60 or 90 grains of pot. iod. a day that will melt like April snow by increasing the dose. I

have at present a case of tertiary throat lesion in which 90 grains gave no result, but 150 is proving rapidly curative. In fact my impression in regard to the use of pot. iod. in syphilis is that we should do away with the idea of a dose, and increase our remedy until we get the desired result. For the rapid cure of dangerous lesions pot. iod. is better than mercury in my estimation, but I do not look upon it as preventive of their return. Consequently after they are under control, mercury takes the first place in our therapeutic measures. The iodide is also a depressant, while mercury in the usual specific dose is a decided tonic.

The other points in these cases have been so thoroughly and lucidly put forth by Dr. Williams that I will not take them up.

